# Perinatal death triples the prevalence of postpartum depression among women in Northern Uganda: A community-based cross-sectional study

**DOI:** 10.1371/journal.pone.0240409

**Published:** 2020-10-13

**Authors:** Anna Agnes Ojok Arach, Noeline Nakasujja, Victoria Nankabirwa, Grace Ndeezi, Juliet Kiguli, David Mukunya, Beatrice Odongkara, Vincentina Achora, Justin Bruno Tongun, Milton Wamboko Musaba, Agnes Napyo, Vivian Zalwango, Thorkild Tylleskar, James K. Tumwine

**Affiliations:** 1 Faculty of Health Sciences, Department of Nursing and Midwifery, Lira University, Lira, Uganda; 2 Department of Psychiatry, School of Medicine, Makerere University College of Health Sciences, Kampala, Uganda; 3 Department of Epidemiology and Biostatistics, School of Public Health, Makerere University College of Health Sciences, Kampala, Uganda; 4 Department of Paediatrics and Child Health, School of Medicine, Makerere University College of Health Sciences, Kampala, Uganda; 5 Department of Community Health and Behavioural Sciences, School of Public Health, Makerere University College of Health Sciences, Kampala, Uganda; 6 Sanyu Africa Research Institute, Mbale, Uganda; 7 Department of Paediatrics and Child Health, Gulu University, Gulu, Uganda; 8 Department of Obstetrics and Gynaecology, Gulu University, Gulu, Uganda; 9 Department of Paediatrics and Child Health, University of Juba, Juba, South Sudan; 10 Faculty of Health Sciences, Department of Obstetrics and Gynaecology, Busitema University, Mbale, Uganda; 11 Faculty of Health Sciences, Department of Public Health, Busitema University, Tororo, Uganda; 12 Centre for International Health, University of Bergen, Bergen, Norway; University of Mississippi Medical Center, UNITED STATES

## Abstract

**Introduction:**

Deaths during the perinatal period remain a big challenge in Africa, with 38 deaths per 1000 pregnancies in Uganda. The consequences of these deaths can be detrimental to the women; some ending up with postpartum depression. We examined the association between perinatal death and postpartum depression among women in Lira district, Northern Uganda.

**Methods:**

We conducted a community-based cross-sectional study of 1,789 women. Trained research assistants screened women for postpartum depressive symptoms on day 50 postpartum using the Edinburgh postpartum depression scale (EPDS). Socio-demographic, economic, birth and survival status of the neonate were collected during pregnancy and within one week postpartum. We used generalized estimating equation for the Poisson family with a log link using Stata to estimate the prevalence ratio of the association between postpartum depressive symptoms (EPDS scores ≥14) and perinatal death. Mothers who lost their babies between 7–49 days postpartum were excluded.

**Results:**

Of the 1,789 participants symptomatically screened for postpartum depression, 377 (21.1%) [95% confidence interval (95%CI): 17.2%, 23.0%] had probable depressive symptoms. The prevalence of postpartum depressive symptoms among the 77 women who had experienced perinatal death (37 stillbirths and 40 early neonatal deaths (≤7 days of life)) was 62.3% [95% CI: 50.8%, 72.6%] compared to 19.2% [95% CI: 17.4%, 21.2%], among 1,712 with live infants at day 50 postpartum. Women who had experienced a perinatal death were three times as likely to have postpartum depressive symptoms as those who had a live birth [adjusted prevalence ratio 3.45 (95% CI: 2.67, 4.48)].

**Conclusions:**

The prevalence of postpartum depressive symptoms, assessed by EPDS, was high among women who had had a perinatal death in Northern Uganda. Women experiencing a perinatal death need to be screened for postpartum depressive symptoms in order to intervene and reduce associated morbidity.

## Introduction

Postpartum depression is a public health problem worldwide, with adverse health consequences to the mother and her family. The burden of postpartum depression varies across countries. A systematic review and metanalysis reported a pooled prevalence of 18.7% with a 95% confidence interval (95% CI) of 17.8–19.7% [1] among women in low- and middle-income countries and a considerably lower prevalence in high-income countries, only 9.5% (95% CI 8.9–10.1) [1].

Postpartum depression is a mood disorder with clinical manifestations which include: inability to sleep, sleepiness, mood swings, change in appetite, fear of harm, sadness, excessive crying, feeling of doubt, guilt and helplessness, difficulty concentrating and remembering, loss of interest in hobbies and usual activities and recurrent suicidal thoughts [2]. Postpartum depression is different from postnatal blues, in that postnatal blues set in within 2–3 days and resolve by 10 days postpartum [3].

In recent years, postpartum depression has drawn public attention because of the negative effects on the affected women which include woman’s social and occupational functioning [4,5], physical health [6], relationship with her spouse, quality of life [5,7], and her long term emotional balance [8]. Although some women are reported to improve from postpartum depressive symptoms, a considerable proportion may experience chronic mental health problems [9–11]. Several factors have been associated with the development of postpartum depression. These can be classified into “biological (change in hormones, age of mother), physical (chronic health problems), psychological (prenatal anxiety, stress, lack of social support, poor marital relationship, stressful life events), obstetrics/pediatrics (unwanted pregnancy, parity, history of loss of pregnancy and poor infant health), and socio-cultural (status of mother, polygamy and poverty)” [12,13]. Evidence associates perinatal death to existence of postpartum depression [14].

Globally, about five million pregnancies end in perinatal deaths yearly with the majority (98%) occurring in sub-Saharan Africa and Asia [15–17]. Perinatal death is often defined as the birth of a stillborn baby from 28 weeks of pregnancy or an early neonatal death, death of a neonate within 7 days of life [17]. Perinatal death impacts negatively on the psycho-social outcome of the affected women [18]. She faces adverse psychological effects which has been noted to progress to the following pregnancy [19]. Women with a perinatal death have been reported to lack psychosocial support. Moreover psychosocial support has been shown to reduce postpartum depression [20].

The frequency of perinatal death in Northern Uganda is estimated to be similar to the national average which is estimated to be 40 deaths per 1000 pregnancies [21]. This is considerably higher than the target of 12 or fewer in the Every Newborn Action Plan (ENAP) [22]. In 2016/2017 Lira district had 27 infant deaths per 1000 deliveries in health facilities alone. Infant death at home and in the community, could be much higher [23], but vital registration is lacking in the area.

Although extensive studies have been conducted on postpartum depression [24–29], few have focused on women who have experienced a perinatal death [14,30–32]. In our study context, the association between perinatal death and postpartum depression has not been established making this evidence scarce in Northern Uganda. Postpartum depression as a result of perinatal death is not addressed in the Ugandan healthcare guidelines. Findings of our study play a role for public health interventionists in focusing initiatives on the most affected women. It is against this background that we examined the prevalence and association between perinatal death and postpartum depression among women in Lira, a district in Northern Uganda.

We hypothesized that women with perinatal death in Lira District were more likely to screen positive for postpartum depression than women without perinatal death.

## Materials and methods

### Study setting

The study was conducted in the rural district of Lira in Northern Uganda. The area is inhabited by the Langi ethnic group. The main economic activity is subsistence farming. The district has three administrative counties, 13 sub-counties, 89 parishes and 751 villages. The district had a population of about 410,000 in 2014 [33] served by 31 health care facilities including 1 referral hospital, 3 Health Centers with a surgical wing (HC IV), 17 Health Centers with maternity (HC III) and 10 Health Center II (HC II, dispensary). This study was conducted in Erute-North county, and covered Aromo and Agweng and Ogur sub-counties. The population of women in the reproductive age for these sub-counties was about 15,000 as of 2016/2017 (Unpublished data). These sub-counties were chosen based on the poor maternal and perinatal indicators and the location in a rural hard-to-reach area of Lira district. For instance, fewer mothers give birth at a health facility, 67% [34] compared to the national average of 76% [21]. Northern Uganda was devastated by armed insurgency, under the Lord’s Resistance Army (LRA) for more than 20 years (1987–2006) that resulted in immense suffering from rape, abductions, massacres, maiming and other heinous atrocities [35], insecurity and displacement of people in camps. The insurgency weakened social and economic services including health. This study was conducted 10 years post-war period, when resettlement had taken place and the region was undergoing reconstruction and rehabilitation.

### Study design

This was a cross-sectional study nested in the Survival Pluss trial; a cluster randomized community-based trial (NCT0260505369). In this paper, we compared postpartum depressive symptoms among women with a perinatal death to those with children alive at 50 days postpartum.

### Study population

The participants in this study were women who had been enrolled in the community-based, community-randomized trial, who gave birth at 28 weeks of gestation or more and were available 50 days postpartum. The enrollment started by visibly pregnant women being identified by a community female volunteer resident in the village. A research assistant accompanied by the community volunteer subsequently visited the pregnant woman in her home to confirm that they were at least 28 weeks pregnant. The pregnant woman was consented and consecutively enrolled in the trial when she fulfilled the eligibility criteria and excluded if she had plans of moving away from study area within 6 months. The trial enrolled participants between January 2018 and November 2018. The participants from the trial were included in this study if they were available at birth, 7 and 50 days postpartum.

### Sample size estimation

For this study, we estimated the required sample size of 1789 using a formula by Fleiss for continuity correction [36]. This estimation factored in 0.05 alpha, 80% power and 10% non-response. We assumed that 43% were depressed without perinatal death [25] and that 50% had postpartum depression with perinatal death.

### Study variables

The study variables were collected simultaneously with that of the clinical trial. The outcome variable was postpartum depression assessed by EDPS defined as a mood disorder that occurs within four weeks postpartum. Postpartum depression has clinical manifestations [2] which are detected by EPDS, a 10-question standard tool [37]. This tool is currently the most widely studied and translated instrument for screening for postpartum depression. It is a usually self-administered questionnaire comprising of 10 questions, each with four possible answers rated from 0 to 3 (typically: Yes, most of the time; Yes, some of the time; Not very often; No, never), and the scale has a maximum score of 30 with a recommended cut off score of 10. It can also be administered in an interview and the self-completed EPDS and directed interview EPDS are equivalent screening techniques for postpartum depression [38]. The Edinburgh postnatal depression scale showed acceptable accuracy in low and middle income countries [39]. It also had a sensitivity and specificity of 68% and 93% at cut off score ≥12 from 14 validation studies conducted in seven African countries [40]. The screening was done on day 50 postpartum which is the ideal period for collecting data on postpartum depression [41,42]. During the interview, there was no interruption from the family members. This is because four (4) visits had already been made at the same home (on the day of recruitment, at birth, day 7 and 28). Therefore, at this 5^th^ visit, the family members were aware of the study and the nature of interaction. Family members especially partners excused themselves, gave privacy and physical space to women. Children were mostly interested in the equipment for measurement such as weighing scales and length boards. They did not keep around their mother as she was interviewed.

Postnatal depression can be detected any time after 4 weeks though it can also persist for up to 12 months or more [11]. It is also known that 50 days postpartum postnatal blues will have resolved for all women after delivery. Different EPDS cut-off scores can be used to classify depression symptoms. The most common cut-off scores are: ≥10, ≥13 and ≥14 out of 30. The conventional interpretation of the cut-off scores are: ≥10 is often interpreted as a woman with “possible depression”, ≥13 a woman with a “fairly high possibility of depression” and ≥14 a woman with “probable depression” [42]. We used the cut-off score of ≥14 in our analysis in order to increase specificity.

Perinatal death was the main exposure variable. We defined perinatal death as birth of a stillborn baby from at least 28 weeks pregnancy or more or an early neonatal death, death of a newborn within 7 days of life [17]. Birth and survival status of the neonate were collected at birth and on day 7 postpartum using standard questionnaires. Fifteen mothers had lost their babies after day 7 but before day 50 and these were not included in the analysis. Participants whose babies died between 7–50 days could not be included in the non-exposed group because their risk of depression does not reflect that of the source population. Losing a child at whatever age places one at a higher risk of depression compared to the general population [14].

Co-variates considered were: 1) Maternal age recorded in completed years and later categorized as ≤19 years, between 20 and 34, and ≥35 years. 2) Education collected as completed years of education and later categorized as no education, primary education only and secondary education or higher. 3) Parity was collected as number of children the woman had ever given birth to. It was categorized as first pregnancy (prime para), 1–4 children, ≥ 5 children during analysis. 4) Marital status was collected as single, married, cohabiting and divorced and later re-grouped into married or not married. 5) Socio-economic status was assessed by the use of an asset-based index, using principal component analysis [43]. This ‘wealth index’ was computed using the first principal component and based on the availability of 9 kinds of household assets. The wealth index was later collapsed into three groups; lowest 40%, middle 40% and top 20%. 6) Place of delivery was recorded as government hospital, health center, private hospital/clinic, drug shop, traditional healer, home, and on the way to hospital and later categorized as health facility delivery or home (= elsewhere). 7) Effect of the parent trial was recorded as control arm or intervention arm, respectively. All these covariates except place of delivery were collected during recruitment. All the data collection tools were translated into *Lango*, back translated into English, pretested, and adjusted where necessary. We collected data between January 2018 and March 2019. A team of 42 research assistants fluent in the local language *Lango* collected the data in a face-to-face interview conducted at the woman’s home. Data was collected using android-based mobile application (Open Data Kit, https://opendatakit.org) and uploaded onto a password protected server.

Research assistants were university graduates, trained on study protocol and ethics. We closely supervised the data collectors. Data was checked for completeness and consistency daily before submission.

### Data analysis

When data collection was complete, data was transferred to Stata version 14.0 (StataCorp; College Station, TX, USA) for cleaning and analysis. We present means and standard deviations for continuous data, and proportions with confidence intervals for categorical data. The prevalence of postpartum depression was estimated as a proportion of women who scored ≥14 in EPDS. In bivariable analysis, we used generalized estimating equation for the Poisson family to test the association of the following factors with postpartum depression: perinatal death, maternal age, level of education, wealth index, marital status, parity, multiple partners of the husband and place of birth.

We adjusted for the following factors, previously shown to be associated with postpartum depression: maternal age [24,26], maternal education [44], parity [25,45], wealth status [44,46], marital status [27] and place of birth [47]. We fitted a model during the multivariable analysis using generalized estimating equation for the Poisson family, with a log link while considering the cluster effect. We assessed all the variables in the model for collinearity. Collinearity was considered if the variables had a variance inflation factor (VIF) greater than 10. In situations of collinearity, we retained the variable with greater biological plausibility. We also assessed for effect modification (potential interaction) of the association between perinatal death and postpartum depression by wealth quintile.

### Ethical considerations

We obtained ethical approval of the study from Makerere University School of Medicine Higher Degrees Research Ethics Committee (REC REF 2017–171), the Uganda National Council for Science and Technology (HS356ES). The parent trial was registered with Clinical Trial.gov and the Regional Committee for Medical and Health Research Ethics, Norway (2017/2079/REK vest). Permission was obtained from the Uganda Ministry of Health and Lira District Local Government. Written informed consent was obtained from all participants after a detailed explanation of the study and purpose. During training of research assistants, the issue of confidentiality, respect for participants, the right of the participants to withdraw their participation from the study at any time without any penalty were emphasized. We used identification numbers instead of names to conceal identity of participants. We obtained permission to conduct the study from community leaders. Women who scored ≥14 in EPDS were referred for further clinical assessment and management by a psychiatrist at the referral hospital in the district.

## Results

### Characteristics of the study population

**Fig 1:**
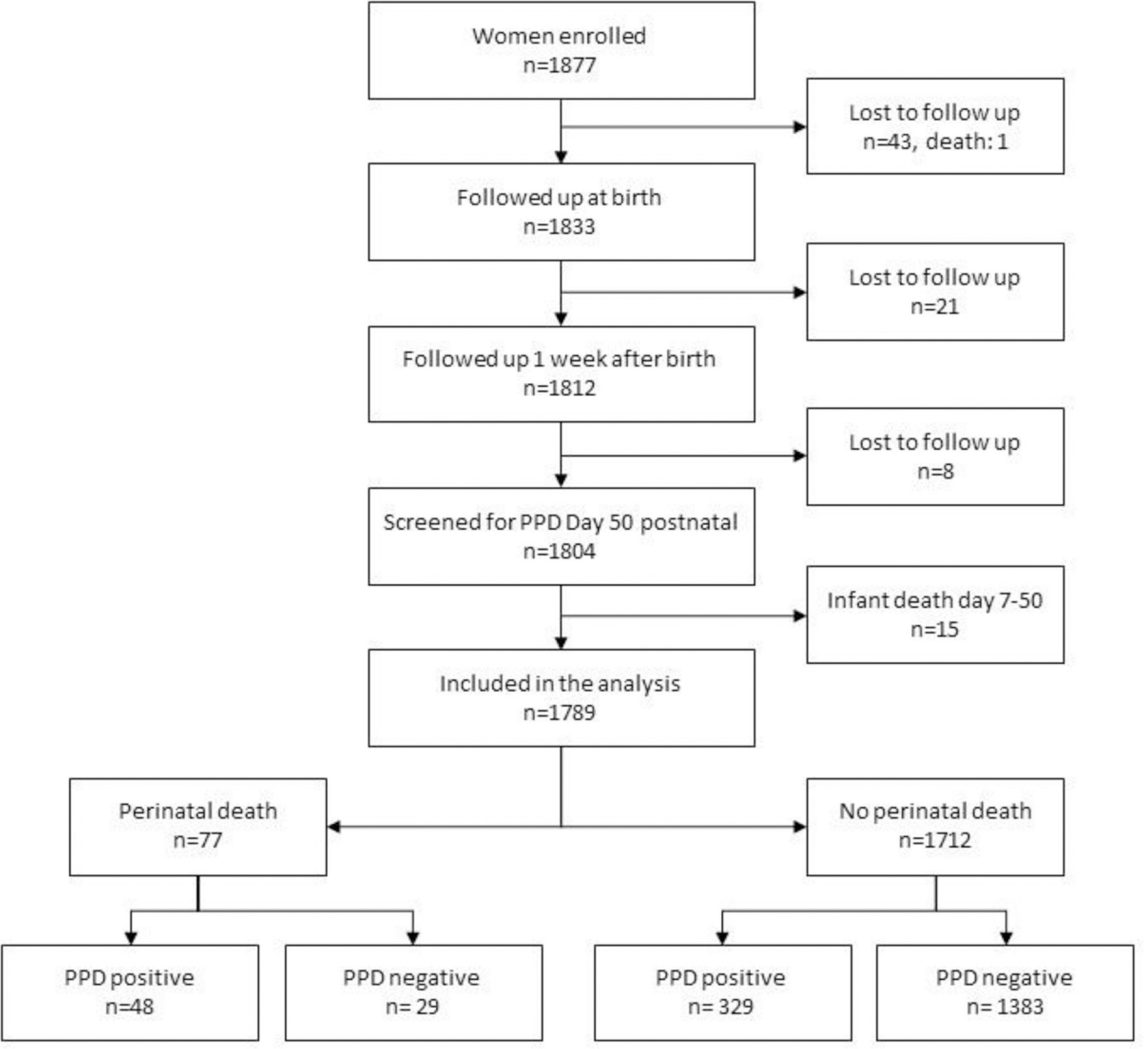
Flow chart of study participants enrolled, followed up and screened for postpartum depressive symptoms.

We present the results for the 1,789 participants who were screened for postpartum depressive symptoms on day 50 postpartum, (Fig 1).

The average maternal age (±SD) was 25 (± 7) years with a range of 12 to 47. Most women had primary education, 77.8% [95% CI: 75.8%, 79.7%]. They had a mean parity of 3 (range 0 to 12). Most of the participants were married, 91.3% [89.9%, 92.3%]. There were 77 (4.3%) perinatal deaths, of whom 37 were stillbirths and 40 were early neonatal deaths from the 1,789 women participants. Out of the 1,789 study participants screened for postpartum depressive symptoms, 377 (21.1%) [95%CI: 17.2%, 23.0%] had “probable depression”(EPDS cut-off score ≥14), 465 (26%) [95% CI: 24.0, 28.1] had a “fairly high possibility of depression” (EPDS cut off score ≥13) and 749 (41.9%) [95% CI: 39.6, 44.2] had “possible depression” (EPDS cut off score ≥10). Table 1.

**Table 1:** Maternal characteristics of women in Lira, Uganda by EPDS Scores and crude prevalence ratio (PR) and adjusted PRs of postpartum depressive symptoms and perinatal death among 1789 women in Lira, Northern Uganda.

	Total N = 1789 n (%)	EPDS Scores ≥14 N = 377 n (%)	EPDS Scores < 14 N = 1412 n (%)	Crude Prevalence Ratio (PR) [95% CI]	Adjusted Prevalence Ratio (PR) [95% CI]
**Perinatal death**					
**No**	1712 (95.7)	329 (87.3)	1383 (98.0)	1	1
**Yes**	77 (4.3)	48 (12.7)	29 (2.0)	3.15 [2.45, 4.04]	**3.45 [2.67, 4.48]**
**Maternal age**					
**≤19 years**	483 (27.0)	78 (20.7)	405 (28.7)	1	1
**20–34**	1124 (62.8)	258 (68.4)	866 (61.3)	1.42 [1.14, 1.78]	1.27 [0.93, 1.76]
**≥35 years**	182 (10.2)	41 (10.9)	141 (10.0)	1.42 [1.04, 1.94]	1.17 [0.74, 1.88]
**Education level**					
**None**	233 (13.0)	54 (14.3)	179 (12.7)	1	1
**Primary**	1392 (77.8)	287 (76.1)	1105 (78.3)	0.88 [0.71, 1.09]	0.96 [0.78, 1.18]
**Secondary or higher**	164 (9.2)	36 (9.6)	128 (9.0)	0.90 [0.65, 1.25]	1.04 [0.73, 1.48]
**Parity**					
**Less than 1**	503 (28.1)	83 (22.0)	420 (29.8)	1	1
**Between 1–4**	913 (51.0)	208 (55.2)	705 (49.9)	1.40 [1.13, 1.74]	1.35 [1.02, 1.79]
**5 and above**	373 (20.9)	86 (22.8)	287 (20.3)	1.42 [1.09, 1.86]	1.40 [0.92, 2.12]
**Marital status**					
**Single**	156 (8.7)	39 (10.3)	117 (8.3)	1	1
**Married**	1633 (91.3)	338 (89.7)	1295 (91.7)	0.83[0.58, 1.18]	0.75 [0.54, 1.04]
**Wealth index**					
**Lowest 40%**	802 (44.8)	176 (46.8)	628 (44.3)	1	1
**Middle 40%**	628 (35.1)	131 (34.7)	497 (35.2)	0.93 [0.79, 1.10]	0.89 [0.77, 1.05]
**Top 20%**	359 (20.1)	70 (18.5)	289 (20.5)	0.89 [0.72, 1.10]	0.79 [0.64, 0.98]
**Place of birth**					
**Home**	575 (32.1)	123 (32.6)	452 (32.0)	1	1
**Health facility**	1214 (67.9)	254 (67.4)	960 (68.0)	0.99 [0.90, 1.09]	1.03 [0.86, 1.24]
**Effect of the trial**					
**Control arm**	841 (47.0)	166 (44.0)	675 (47.8)	1	1
**Intervention arm**	948 (53.0)	211 (55.0)	737 (52.2)	1.10 [0.83, 1.45]	1.09 [0.82, 1.44]

### Prevalence of postpartum depressive symptoms

**Fig 2:**
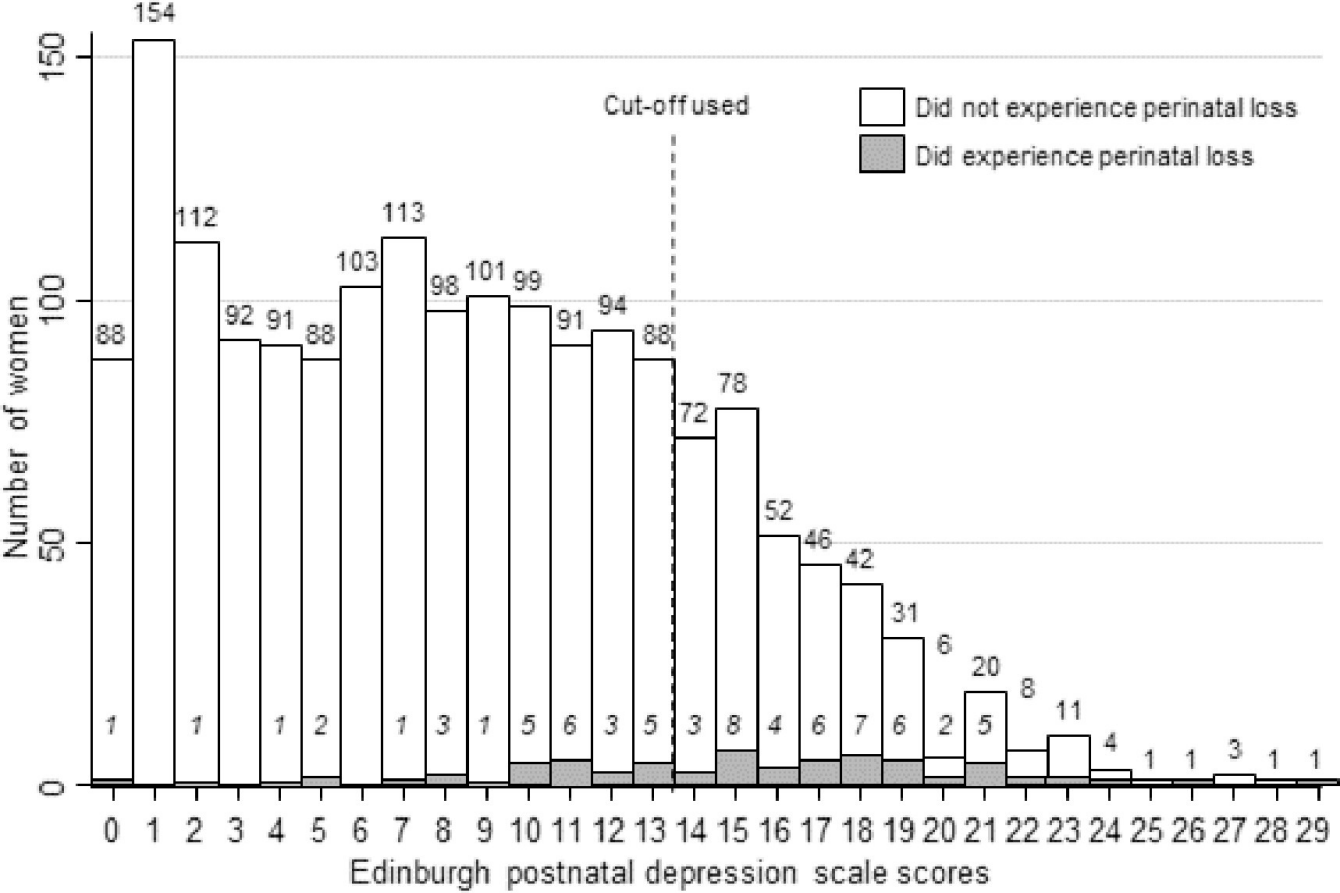
Scores of the Edinburgh postnatal depression scale among participants 50 days postpartum in Lira, Northern Uganda, 2018–2019 (N = 1789).

**Fig 3:**
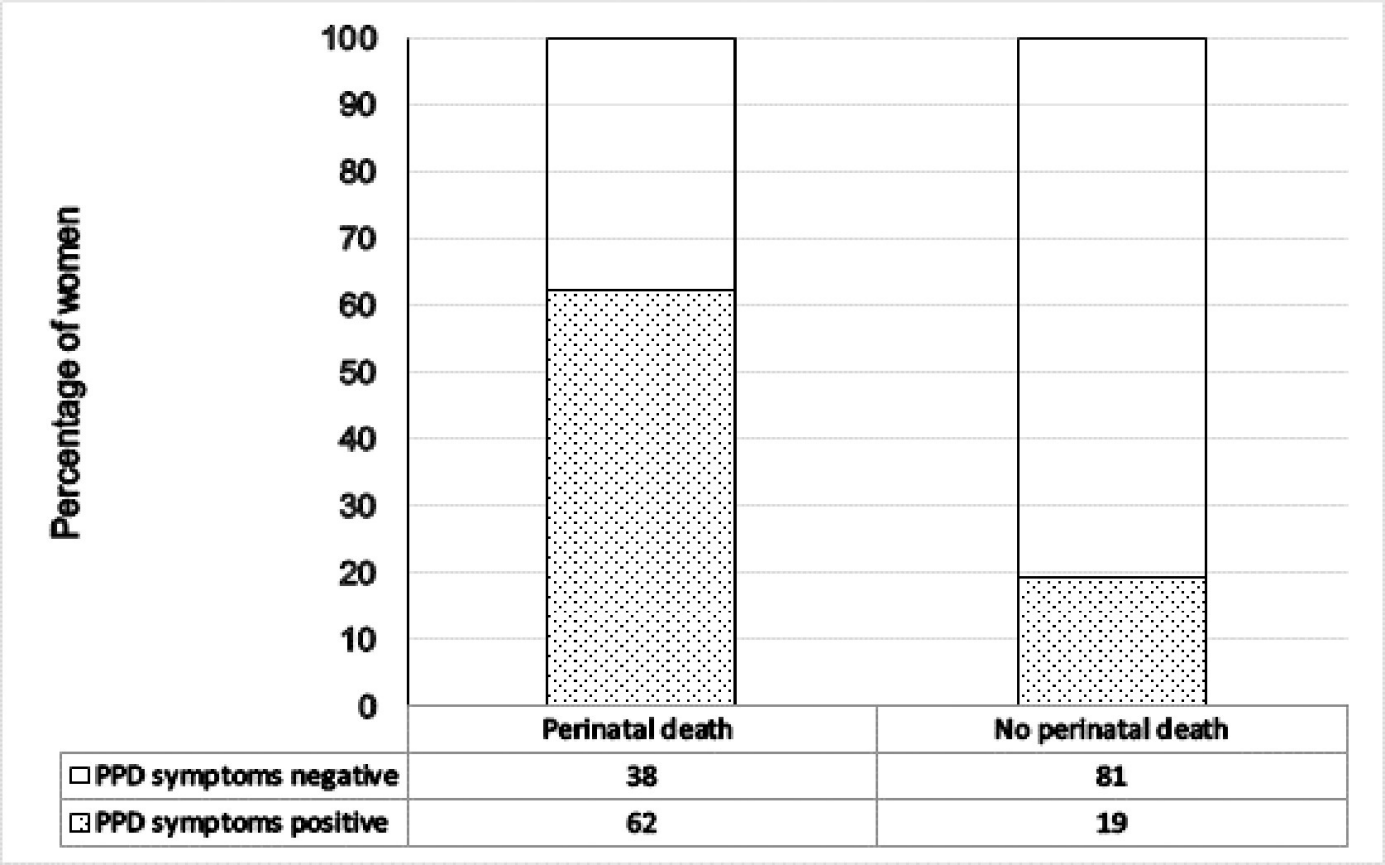
Prevalence of postpartum depressive symptoms (EPDS scores ≥14) among women who had perinatal death or live infants in Lira, Northern Uganda (N = 1789).

The mean score was 8.5 ±6, with a range of 0 to 29 (Fig 2). Seventeen women reported having frequent suicidal thoughts. From the 77 women who experienced perinatal death, 48 (62.3%) [95%CI: 50.81%, 72.61%] screened positive for “probable depression” (EPDS scores ≥14) compared to 329 (19.21%) [95% CI: 17.42%, 21.15%] out of 1712 women with live infants (Fig 3).

### Factors associated with postpartum depressive symptoms

Women who had experienced a perinatal death were three times as likely to have probable postpartum depression as those who had had a live infant at day 50 postpartum (adjusted prevalence ratio 3.45, [95% CI: 2.67, 4.48]), Table 1. Generally, experiencing perinatal death was associated with screening positive for postpartum depressive symptoms with EPDS using cut-off score of 14 in both crude and adjusted analysis. We controlled for maternal age, education status, parity, wealth status, place of childbirth, and effect of the trial. When we stratified the data by wealth quintile, the prevalence ratio (PR) between postpartum depression and perinatal death did not vary significantly across the different categories of wealth index (Table 2).

**Table 2:** Adjusted prevalence ratio between postpartum depression and perinatal death stratified by wealth index.

	Lowest 40% N = 802	Middle 40% N = 628	Top 20% N = 359
	Adjusted Prevalence Ratio (PR) [95% CI]	Adjusted Prevalence Ratio (PR) [95% CI]	Adjusted Prevalence Ratio (PR) [95% CI]
**Perinatal death**			
**No**	1	1	1
**Yes**	**3.21 [2.16, 4.77]**	**3.88 [2.79, 5.39]**	**3.28 [1.98, 5.45]**
**Maternal age**			
**≤19 years**	1	1	1
**20–34**	1.50 [1.04, 2.18]	1.13 [0.62, 2.07]	1.25, [0.40, 3.85]
**≥35 years**	1.27 [0.58, 2.78]	0.97 [0.45, 2.10]	1.76 [0.44, 3.85]
**Education level**			
**None**	1	1	1
**Primary**	0.86 [0.64, 1.16]	0.89 [0.65, 1.22]	2.28 [0.88, 5.88]
**Secondary or higher**	0.98 [0.56, 1.73]	0.89 [0.44, 1.79]	2.55 [0.93, 6.97]
**Parity**			
**Less than 1**	1	1	1
**Between 1–4**	0.99 [0.71, 1.37]	1.57 [0.89, 2.77]	2.68 [1.22, 5.89]
**5 and above**	1.14 [0.61, 2.13]	1.48 [0.82, 2.69]	2.19 [0.72, 6.69]
**Marital status**			
**Single**	1	1	1
**Married**	0.72 [0.48, 1.08]	0.74 [0.45, 1.24]	0.84 [0.33, 2.08]
**Place of birth**			
**Home**	1	1	1
**Health facility**	0.99 [0.77, 1.27]	1.12 [0.79, 1.58]	0.96 [0.65, 1.42]
**Effect of the trial**			
**Control arm**	1	1	1
**Intervention arm**	1.10 [0.79, 1.54]	1.11 [0.77, 1.58]	1.04 [0.71, 1.52]

## Discussion

This community-based study in Northern Uganda including over 1700 women who had given birth and were interviewed at day 50 postpartum shows, first that women who experienced a perinatal death were three times as likely to have a ‘probable depression’ as those who had had a live baby at day 50 and second, that 21.1% (95%CI: 17.2, 23.0) of the women had a ‘probable depression’ with the assessment method used.

The result that women with a perinatal loss had an increased risk of postnatal depression could be interpreted in light of the theory of chronic sorrow proposed by Eakes, Burkes and Hainsworth [48]. According to the theory, significant losses such as pregnancy loss or loss of an infant creates a disparity between an actual reality and one’s expectation [48]. Because of this disparity, these women continue to experience periodic recurrence of pervasive sadness, sorrow or other grief-related feelings which share similarities with postpartum depression symptom [2]. Therefore, it is plausible that women who had had a perinatal death in our study were more likely to experience postpartum depression compared to their counterparts. Our study findings are consistent with findings from Bangladesh and Malaysia [14,30,32].

The association between perinatal death and postpartum depression was similar across socio-economic strata. Some studies, on the other hand, have reported that poorer women were more likely to suffer from postpartum depression [45,49]. The inconsistency [45,50] could be due to several things; one is the difference in the wealth assessment. In our study, we used a relative index and not an absolute wealth index [43]. Since the poverty level in Northern Uganda is considerably higher than the national average (32.5% compared to 21.4%) [51], it could be that our participants overall were poor, and the relative difference did not play any role.

The prevalence of postpartum depression among women with live infants was 19%, similar to what was found in a recent systematic review for low and middle-income countries [1].

When comparing prevalences from different places, countries or even continents, there are a number of issues to keep in mind.

The first thing to bear in mind is the choice of screening tool. We used the Edinburgh postnatal depression scale (EPDS) but there are other scales in use [27,52].

In our study we were particularly interested in the women who had a perinatal death and the other women can be said to be a kind of ‘control group’ when it comes to the use of EPDS. In spite of the fact that the EPDS tool has not been validated in the study area, we are confident that the difference between the groups of women with and without a perinatal death is a real difference.

The second thing to bear in mind is the cut-off used. Generally speaking in EPDS, the lower you set the cut-off, the higher the proportion of women scoring above the cut-off. It is therefore important to compare studies using the same cut-off.

Whereas these findings call for depression screening of both women who had perinatal deaths and those with live infants during the postnatal care visit, Uganda’s health system has been reported to have limited maternal mental health services [53]. This signifies minimal maternal evaluation for postpartum depression. Therefore, there is a need for incorporation of postpartum depression screening in postnatal care at all levels of the health care system in addition to training of midwives on depression detection during the postnatal visit at 6–8 weeks.

### Strengths and limitations

Our study had enough power to assess the association between perinatal death and postpartum depression. It was a community-based cross-sectional study, where we were able to include participants who gave birth at home and who could have been missed in a health facility-based study. Nesting it in a follow-up study was a strength since the research team were able to screen all the women at the same time, 50^th^ day after child birth. Information bias on reporting perinatal death was minimized by the follow-up visit within 24 hours of child birth and 7^th^ day postpartum. The screening was conducted on day 50 (week 7); an ideal period that is known for postpartum depression detection. Contrasting two groups of women provides an internal consistency test of the assessment tool.

Some factors such as lack of social support, intimate partner violence/marital conflict and mistreatment of women during childbirth were not assessed, yet they have been reported to be independent predictors of postpartum depression in previous studies. The Edinburgh postnatal depression scale was not validated in this population. Examining the incidence of postpartum depression symptoms in this study was not possible because follow-up in the parent study did not start with a cohort free of the depressive symptoms.

## Conclusions

The prevalence of postpartum depressive symptoms, assessed by EPDS, was high among women who had had a perinatal death in Northern Uganda. Women who experience a perinatal death need to be screened for postpartum depressive symptoms in order to intervene and reduce associated morbidity.

## Supporting information

S1 File Data set PDPPD. (DAT)
